# RNA Expression Analysis of Mycobacterial Methyltransferases Genes in Different Resistant Strains of *Mycobacterium tuberculosis*

**DOI:** 10.52547/ibj.26.3.240

**Published:** 2022-02-12

**Authors:** Samira Tarashi, Mohammad Saber Zamani, Golnaz Bahramali, Andrea Fuso, Farzam Vaziri, Morteza Karimipoor, Abolfazl Fateh, Seyed Davar Siadat

**Affiliations:** 1 Department of Mycobacteriology and Pulmonary Research, Pasteur Institute of Iran, Tehran, Iran;; 2 Microbiology Research Center, Pasteur Institute of Iran, Tehran, Iran;; 3 Immunoregulation Research Center, Shahed University, Tehran, Iran;; 4 Department of Hepatitis and AIDS, Pasteur Institute of Iran, Tehran, Iran;; 5 Viral Vaccine Research Center, Pasteur Institute of Iran, Tehran, Iran;; 6 Department of Experimental Medicine, Sapienza University of Rome, Italy;; 7 Molecular Medicine Department, Biotechnology Research Center, Pasteur Institute of Iran, Tehran, Iran

**Keywords:** Drug resistance, Methyltransferases, Mycobacterium tuberculosis, S-adenosylmethionine

## Abstract

**Background:**

Tuberculosis infection still represents a global health issue affecting patients worldwide. Strategies for its control may be not as effective as it should be, specifically in case of resistant strains of *M.tb*. In this regard, the role of mycobacterial MTases in TB infection can be fundamental, though it has not been broadly deciphered.

**Methods:**

Five resistant isolates of *M.tb* were obtained. *M.tb *H37Rv (ATCC 27249) was used as a reference strain. Seven putative mycobacterial MTase genes (*Rv0645c*, *Rv2966c*,* Rv1988*,* Rv1694*, *Rv3919c*, *Rv2756c*, and* Rv3263*) and *Rv1392* as SAM synthase were selected for analysis. PCR-sequencing and qRT-PCR were performed to compare mutations and expression levels of MTases in different strains. The 2^-ΔΔCt^ method was employed to calculate the relative expression levels of these genes.

**Results:**

Only two mutations were found in INH^R^ strain for *Rv3919c *(T to G in codon 341) and* Rv1392 *(G to A in codon 97) genes. Overexpression of *Rv0645c*, *Rv2756c*, *Rv3263*, and *Rv2966c* was detected in all sensitive and resistant isolates. However, *Rv1988* and *Rv3919c* decreased and *Rv1694* increased in the sensitive strains. The* Rv1392* expression level also decreased in INH^R^ isolate.

**Conclusion:**

We found a correlation between mycobacterial MTases expression and resistance to antibiotics in *M.tb *strains. Some MTases undeniably are virulence factors that specifically hijack the host defense mechanism. Further evaluations are needed to explore the complete impact of mycobacterial MTases within specific strains of *M.tb *to introduce novel diagnosis and treatment strategies.

## INTRODUCTION

Tuberculosis is an infectious disease mostly affecting the lungs and is caused by *M.tb*. Historically, TB is defined as one of the most devastating epidemiological concerns in human health. According to the latest WHO report in 2019, 10 million new TB cases and 1.2 million TB-related deaths were estimated^[1]^. The importance of this infection in healthcare settings increases with the appearance of resistant strains, specifically, MDR-TB (resistant to INH and RIF), and XDR-TB (resistant to INH and RIF plus fluoroquinolones and at least one injectable agent, including amikacin, kanamycin, and capreomycin) strains^[2]^. Resistance to a range of antibiotics is an important feature of *M.tb* due to the fact that these pathogens have in-built complex regulation machinery. In 2019, 465,000 individuals were infected with drug-resistant TB^[1]^. Early diagnosis and accurate treatment are two major objectives in TB infection control^[3]^. Therefore, delay in the correct diagnosis of *M.tb *strains and proper treatment are the most significant causes of TB mortality^[4]^. Despite the intense research, the control of TB is still largely suboptimal, and the mechanisms of its progression cannot completely be explained. The methylation process is one of the most prominent regulatory mechanisms that has attracted enormous interests in TB researches^[5]^. 

Some of the microorganisms induce methylation in the genome of the host cells or even their own genome to exacerbate virulence and decrease immune responses^[6,7]^. Although mycobacterial methylation has been addressed, little information is available in drug-resistant *M.tb *strains^[8]^. The process of methylation is catalyzed by different MTases^[9]^. About 121 different MTases have been coded by the genome of *M.tb*, which employs several substrates, including DNA, RNA, protein, intermediates of mycolic acid biosynthesis, and other fatty acids^[10]^. Among them, methylation of DNA and proteins (histones) are known as two most important epigenetic modifications, which notably regulate gene function without any direct modification in the DNA sequence^[11]^. Recently, *M.tb *has been recognized as an epigenome regulator controlling many cellular processes involved in host immune system^[12]^. The inter-talk between epigenetic modifications and *M.tb *is also very remarkable in the pathogenicity^[7]^. About 65% of the *M.tb *genome consists of GC-rich regions; therefore, DNA methylation introduced as a highly efficient mechanism to control gene expression^[10]^. 

Overall, different mycobacterial MTases exert their regulatory function on the host cell (mostly genes involved in immune response) or their own genome to finally exacerbate infection by increasing virulence and decreasing immune reactions^[5]^. Lately, it has been indicated that the methylation at different stages of TB infection, as a potential biomarker, requires further consideration^[7]^, and assessment of mycobacterial MTases may revolutionize the current view on TB infection. It is expected the evaluation of the possible link between different mycobacterial MTases expressions and TB resistance can be helpful for developing some strategies to accurately prevent, treat, and control TB. Accordingly, the aim of the current study was to assess the expression level of mycobacterial MTases in different resistance strains of *M.tb *(sensitive, INH^R^, Rif^R^, MDR, and XDR). The presented data may shed new light on host susceptibility and TB severity.

## MATERIALS AND METHODS

**Mycobacterial strains and DST**

Five phenotypically characterized resistant isolates of *M.tb* were obtained from the Mycobacteriology and Pulmonary Research Department of Pasteur Institute of Iran, Tehran. These strains included sensitive, INH^R^, Rif^R^, MDR, and XDR. H37Rv strain (ATCC 27249) was used as a reference strain. All isolates were grown to mid log phase in Middlebrook 7H9 medium supplemented with 0.05% Tween 80, 10% (v/v) oleic acid, albumin, dextrose, catalase^[13]^. The proportion method was performed using the Lowenstein-Jensen medium^[14]^. The critical concentrations of DST were 0.2 μg/mL (INH), 40 μg/mL (RIF), 2.0 μg/mL (ethambutol), 4.0 μg/mL (streptomycin), 40 μg/mL (kanamycin), 30 μg/mL (amikacin), 40 μg/mL (capreomycin), and 4.0 μg/mL (ofloxacin) for phenotypic confirmation^[15]^. All experiments were conducted in accordance with the guidelines approved by the Centers for Disease Control and Prevention (USA)^[16]^. 

**Selection of mycobacterial MTase genes**

Putative mycobacterial MTase genes were selected based on searching from the TubercuList database (http://genolist.pasteur.fr/TubercuList/). The important mycobacterial MTase genes and their related functions are presented in Table 1. After review of MTase genes, we selected seven putative genes (*Rv0645c*, *Rv2966c*,* Rv1988*,* Rv1694*, *Rv3919c*, *Rv2756c*, and* Rv3263 *for analysis. All the selected genes were SAM-dependent mycobacterial MTases. In addition, *Rv1392* as SAM synthase was selected for the evaluation of SAM biosynthesis in different *M.tb *strains^[17]^.

**Table 1:** Mycobacterial MTases

**Mycolic acid MTases**	**DNA ** **MTases**	**RNA ** **MTases**	**Protein ** **MTases**	**Other ** **MTases**
Rv3392c (CmaA1)	Rv1317c (AdaA-AlkA) (Neutralization of mutagenic effect of alkylation stress by demethylation of methylated bacterial DNA)	Rv2118c (tRNA MTases)	**Rv1988** (Methylation of H3 in the host cell)	Rv0447c (UfaA) (tuberculostearic acid MTases)
Rv0503c (CmaA2)	Rv1316c (AdaB/OGT) (Neutralization of mutagenic effect of alkylation stress by demethylation of methylated bacterial DNA)	**Rv2966c** (Methylation of mycobacterial 16SrRNA) (Rv2666c is also a DNA MTase which often methylate the non-CpG regions of host genome) (Rv2966c is a histone MTase in the genome of host cells)	**Rv2966c** (Methylation of H4 and H3 in the host cell)	Rv2952 (Phthiotriol/phenolphthiotriol dimycocerosates MTases)
Rv0645c (MmaA1)	Rv3263 (MamA) (Protection of *M. tuberculosis *in hypoxic status)	Rv2372c (16SrRNA MTase)	Rv1198 (Methylation of H3 in the host cell)	Rv2954c (methylation of phenolic glycolipids)
Rv0644c (MmaA2)	MamB	**Rv1988** (23SrRNA MTase) (Increasing of Macrolide- Lincosamides-Streptomycin resistance)	SET8 (monomethylase H4)	Rv2955c (methylation of phenolic glycolipids)
Rv0643c (MmaA3)	Rv2756c (HsdM)	Rv1694 (23S/16SrRNA MTase) (Increasing of Capreomycin resistance)	SUV39H1 (Methylation of mycobacterial Histone like proteins HupB)	Rv2956 (methylation of phenolic glycolipids)
Rv0642c (MmaA4)	-	Rv3919c (16SrRNA MTase) (Increasing of Streptomycin resistance)	-	-
Rv0470c (PcaA/UmaA2)	-	-	-	-
Rv0469 (UmaA)	-	-	-	-

Multifunctional mycobacterial MTases are shown in bold, and some enzymes affecting the genome of host cells are marked by underline. The other enzymes modify the methylation pattern of mycobacterial genome.

**Evaluation of mycobacterial MTase genes by PCR sequencing**

**Supplementary Fig. 1:**
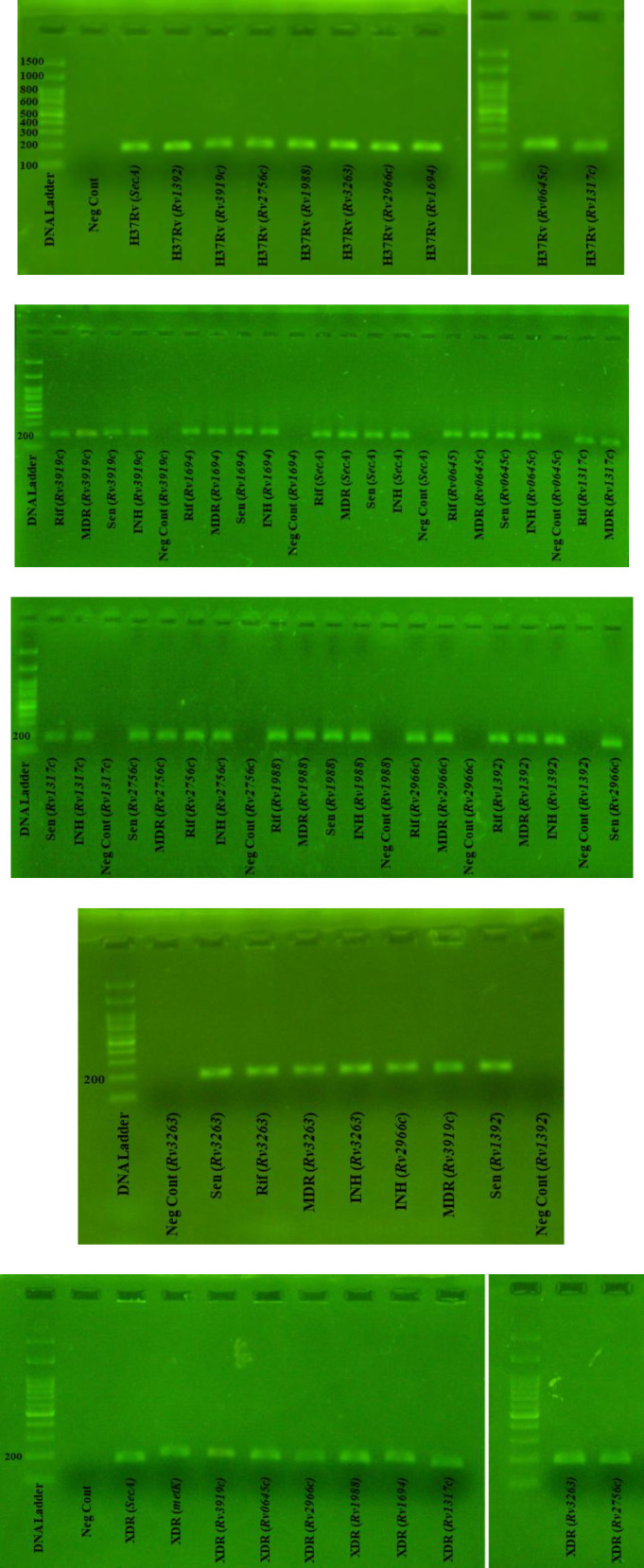
The PCR products of mycobacterial MTases and *Rv1392* genes electrophoresed on 2% (w/v) agarose gel. The related fragments was observed and verified by sequencing. DNA ladder (100 bp); NegCont, negative control.

To confirm the presence of these mycobacterial MTase genes in selected strains, we extracted genomic DNA from the mycobacterial culture in 7H9 media using DNA Technology kit (DNA Technology Co., Moscow, Russia) according to the manufacturer’s instructions. The extracted DNA was dissolved in 30 μl of diethyl pyrocarbonate treated water, and a 10-μl aliquot of total DNA was used for a 25-μl PCR mixture. Table 2 lists the primers employed to amplify the selected regions of mycobacterial DNA in each studied strain. The amplification parameters are shown in the supplementary data (Table S1). The PCR products were analyzed by agarose gel electrophoresis (Fig. S1). Positive and negative controls with or without the genomic DNA of *M.tb *complex were included in each run. Afterward, all the PCR products were purified by PCR purification kit (Thermo Scientific™, USA) and sequenced to evaluate the possible mutations. Raw data was analyzed by MEGA 5.2 software.

**Table 2:** Primers used for PCR and qRT-PCR in this study

**Gene**	**Sequence (5′-3′)**	**Tm**	**Product length (bp)**
*Rv0645c*	F-ACCATATTACGAAGAGTCACA	54.12	195
	R-ACGTCGAGTAGCGTCATC	56.62	
			
*Rv3263*	F-AATTGCTTCAAGTCACCTA	51.65	194
	R-GTTCAGATCGTTCGCATC	53.75	
			
*Rv2756c*	F-AGGATGTCTCGATCTATG	49.67	190
	R-CAGTCTTTGATGTTGAAC	48.62	
			
*Rv2966c*	F-TCGCTATTCAACATCGTGACT	57.5	192
	R-CGCACCGGAGAGACCTAG	58.89	
			
*Rv1988*	F-CGATTCCCTGGCATTACC	55.17	192
	R-AGAAGCGAATTTACATACGA	52.48	
			
*Rv1694*	F-ATATCGTTCCACTGGTGA	52.38	196
	R-AGGAAGTACTCGACATTG	50.87	
			
*Rv3919c*	F-CAGGTAGTTCTCCTAGAA	48.88	188
	R-CTCCATTTCGTCAACTTG	51.07	
			
*Rv1392*	F-GCTGTTTACCAGTGAGTC	52.65	198
	R-TTGGTGATGTCGGCAAAC	55.91	
			
*SecA*	F-CACTACGAGGTCGATCTA	52.13	180
	R-GACGATGTAGTCCTTGTC	51.83	

**Evaluation of mycobacterial MTase genes expression**

The total mycobacterial RNA was isolated using Trizol kit (Plus RNA; Invitrogen, USA) and according to a method described previously^[18]^. Reverse transcription of complementary DNAs and qRT-PCR were performed simultaneously using a QuantiTect SYBR Green RT-PCR Kit (Qiagen, Germany) and a Light Cycler 2.0 instrument (Roche, Switzerland). Each sample was performed in triplicate in a 10-μL capillary tube containing 5 μL of 2× QuantiTect SYBR Green RT-PCR Master Mix, 0.5 μL of a 0.5-μM solution of each primer, 3 μL of RNase-free water, and 1 μL of template RNA (100 ng/μL). The primers used for qRTPCR are listed in Table 2. The assay conditions were represented in supplementary data (Table S2). The *secA* gene was used for normalization as a housekeeping gene. The 2^-ΔΔCt^ method was applied to calculate the relative expression levels of mycobacterial MTase genes^[17]^.

**Statistical analysis**

GraphPad Prism 8 (GraphPad, La Jolla, CA, USA) was used for statistical analyses and drawing graphs. Data were expressed as mean ± SEM. Differences between the two groups were assessed using the nonparametric test, Mann–Whitney U test. A *p *value ≤0.05 was considered statistically significant.

## RESULTS

The susceptibility of all five *M.tb *isolates was confirmed by DST. No growth of sensitive and H37Rv isolates was observed in the Lowenstein-Jensen medium combined with the mentioned antibiotics, i.e. INH, RIF, ethambutol, streptomycin, kanamycin, amikacin, capreomycin, and ofloxacin. The resistance to streptomycin was detected in RIF^R^ isolate, as well as in MDR and XDR isolates. 

**Fig. 1:**
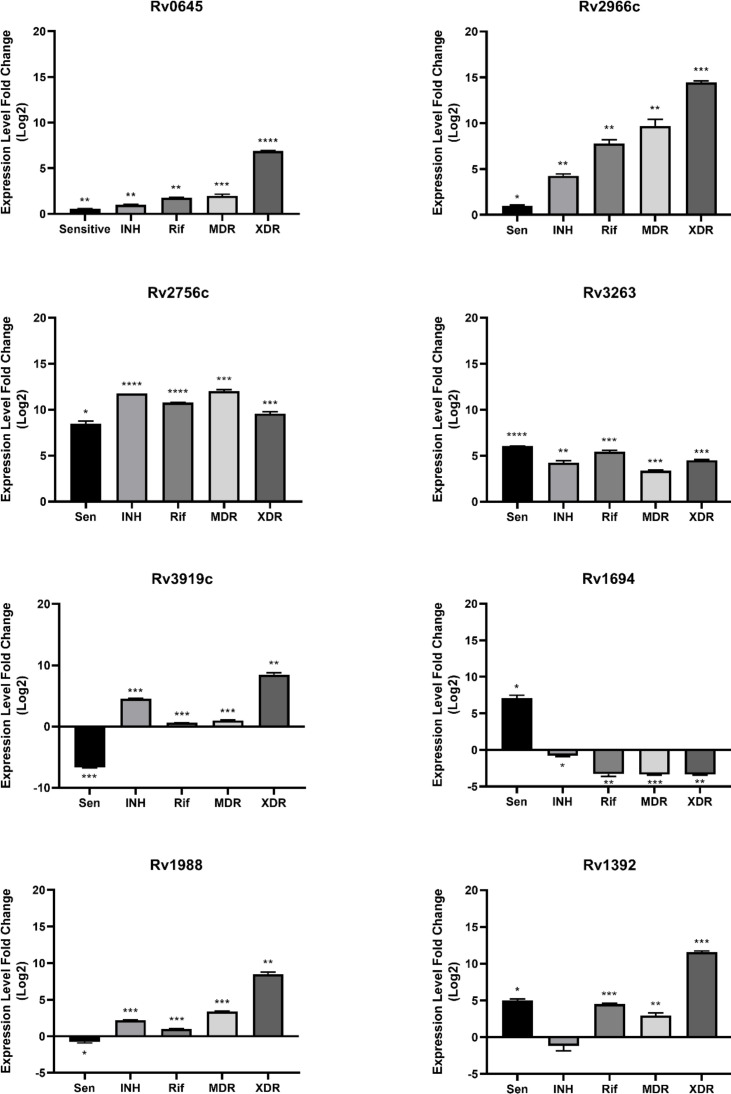
Expression level of mycobacterial MTases and *Rv1392* in different resistant strains of *M.tb*; Mann–Whitney U test.

The important mycobacterial MTase genes are categorized in Table 1 considering their substrate. In the current study, the selection of mycobacterial MTase genes for analysis was based on their category and dependent on SAM. The relative expression level of each evaluated gene compared to H37Rv has been presented in Figure 1. *Rv0645c* was selected as a SAM-dependent mycolic acid MTase, which increased in all sensitive (*p *= 0.006), INH^R^ (*p *= 0.00), RIF^R^ (*p *= 0.007), MDR (*p *= 0.0005), and XDR (*p *< 0.0001) isolates.

The overexpression of a multifunctional mycobacterial MTase gene), *Rv2966c*, foundin sensitive (*p *= 0.02), INH^R^ (*p *= 0.002), RIF^R^ (*p *= 0.002), MDR (*p *= 0.005), and XDR (*p *= 0.0002) isolates. Besides, three genes, including *Rv1988*,* Rv1694*, and* Rv3919c*, were selected from RNA MTase category to be evaluated in antibiotic resistance. The *Rv1988 *has been identified as a histone MTase in host cells during TB infection^[19]^. Its downregulation was shown in sensitive (*p *= 0.02) isolate, and its upregulation was found in INH^R^ (*p *= 0.0008), RIF^R^ (*p *= 0.0005), MDR (*p *= 0.0007), and XDR (*p *= 0.001) isolates. *Rv1694* (*tlyA*) is a 16S rRNA/23S MTase inducing capreomaycin sensitivity, which upregulated only in sensitive (*p *= 0.02) isolate and downregulated in XDR (*p *= 0.002), INH^R^ (*p *= 0.02), RIF^R^ (*p *= 0.009), and MDR (*p *= 0.0006). *Rv3919c* (*gidB*) gene, a mycobacterial 16S rRNA MTase, is involved in the induction of streptomycin resistance in *M.tb*. We observed a mutation in INH^R^ strain for *Rv3919c *(T to G in codon 341). Its expression level showed downregulation only in sensitive (*p *= 0.0003) and upregulation in INH^R^ (*p *= 0.0002), RIF^R^ (*p *= 0.0005), MDR (*p *= 0.0005), and XDR (*p *= 0.001) isolates. *Rv3263* and* Rv2756c* genes were selected for the evaluation of DNA MTases. The activity of *Rv3263* (MamA) has been confirmed in the hypoxic conditions^[20]^. Its upregulation was detected in sensitive (*p *< 0.0001), INH^R^ (*p *= 0.002), RIF^R^ (*p *= 0.0008), MDR (*p *= 0.0007), and XDR (*p *= 0.0006). The expression level of *Rv2756c *(HsdM) increased in all sensitive (*p *= 0.03), INH^R^ (*p *< 0.0001), RIF^R^ (*p *< 0.0001), MDR (*p *= 0.0002), and XDR (*p *= 0.0005) isolates. 

Finally, in addition to mycobacterial MTase genes, *Rv1392* was selected for comparing the SAM biosynthesis in different *M.tb *strains. Rv1392, a SAM synthase, is known as a fundamental member of SAM synthases family^[17]^. A mutation (G to A in codon 97) was detected in INH^R^ strain for this gene. The* Rv1392* expression level decreased only in INH^R^ (*p *= 0.2) and increased in sensitive (*p *= 0.01), RIF^R^ (*p *= 0.0009), MDR (*p *= 0.001), and XDR (*p *= 0.0002) isolates. 

## DISCUSSION

In this study, we evaluated the relationship between mycobacterial MTases expression in different resistant strains of *M.tb*. Also, we compared four categories of mycobacterial MTase genes in five sensitive and resistant strains of *M.tb*. The mycobacterial MTases can be divided into DNA MTases, RNA MTases, protein MTases, mycolic acid MTases, and other MTases^[5]^. These enzymes transfer a methyl (-CH3) group from methyl donors such as SAM to nucleic acids, proteins, lipids, and secondary metabolites. Detection of methylated cytosine in H37Rv and its absence in H37Ra may indicate the possible role of mycobacterial MTases in the pathogenesis^[21]^. More than 1% of human genes have been predicted to encode MTases, while this number has increased to 3% in *M.tb*, in spite of its genome reduction^[5]^. Sharma *et al.*^[22]^ detected 17 putative genes coding for proteins with methylase activity in *M.tb*. The identification of these genes is based on the sequence analysis, SAM binding site prediction, and gene binding domains. 

We reported the overexpression of* Rv0645c *(methoxy mycolic acid synthase 1) as a mycolic acid MTase gene with no mutation in all sensitive and resistant* M.tb *isolates. The upregulation of *Rv0645c* was significantly higher in XDR than other isolates. A study compared differences in the cell wall thickness of sensitive (15.6 ± 1.3 nm), MDR (17.1 ± 1.03 nm) and XDR (20.2 ± 1.5 nm) isolates (*p *< 0.05)^[23]^. This result suggests the possible reason for significant overexpression of *Rv0645c* in XDR in comparison to other strains. The suitable structure of mycolic acid is important in both the permeability of the cell envelope and resistance to antimycobacterial agents. Although various mycolic acid MTases are involved in formation of different mycolic acid structures^[24]^, the exact role of these methylases in the pathogenicity of *M.tb *is unclear yet. Nevertheless, many of the mycolic acid MTase genes are conserved, and no mutation has been detected. In this sense, they may play a critical function in the *M.tb *pathogenicity and have a potential to introduce as a new target in the development of anti-mycobacterial drugs^[25]^. The upregulation of *Rv0645c* has been confirmed during primary infection of macrophages^[26]^. This result suggests that the intracellular *M.tb *actively senses its environment and repairs or synthesizes its cell wall and other components. In addition, a mouse model study showed that the deletion of mycolic acid MTase genes leads to the disruption of the mycolic acid structure, thereby resulting in sensitivity to detergents and causing the general loss of mycobacterial acid-fast feature^[27]^.

Our investigation explored the overexpression of *Rv2756c *and* Rv3263*, mycobacterial DNA MTases, in all sensitive and resistant* M.tb *isolates. DNA methylation epigenetically affects the tolerance, virulence, antibiotic resistance, survival during stress conditions, and intensity of pathogenicity^[8,28]^. An evaluation on DNA methylation diversity in *M.tb *has indicated some mutations in certain mycobacterial DNA MTases such as *Rv2756c *and* Rv3263, *affecting their activity in lineages of *M.tb*^[29]^. The activity of *Rv3263* was detected in all analyzed isolates, except Beijing strains that had high potency to cause active TB and drug resistance. Nevertheless, this fact should not be ignored that 70% of mycobacterial MTases often belongs to a multigene family that may influence the expression level of each other^[10]^. As an example, the *Rv3263* site shares overlapping -10 sigma factor binding site, which is directly bound by the RNA polymerase during the initiation of transcription process and regulates gene expression in response to numerous environmental conditions such as stress and antibiotic resistance^[20]^. However, this document could not establish a direct association between mutation, multigene, and lack of mycobacterial MTase activity, and DNA MTase efficiency has been proved to be particularly variable, despite the presence of their genes^[29]^. A point to the role of different MTases has been highlighted by Grover *et al*.^[10]^ who observed *Rv2756c* only in the pathogenic mycobacteria, reflecting the importance of mycobacterial MTases in the pathogenesis. In general, there is a very limited evidence to clearly explain the cause of such expression modifications of DNA MTases in different resistant and sensitive strains of *M.tb*, and more evaluations are needed to explore their impact. 

Among various groups of mycobacterial MTases, some are multifunctional. *Rv2966c* has been introduced as one of the most important MTases that has been categorized as DNA MTase, protein MTase, and also RNA MTase^[22,30,31]^. We reported the upregulation of *Rv2966c *in the sensitive and drug-resistant mycobacterial strains. Upregulation of this gene appears to be associated with a greater need for the suppression of host gene expression during infection by such mycobacterial strains. Indeed, *Rv2966c* is known as a specific mycobacterial DNA MTase, which is capable of host genes’ nethylation, particularly in non-CpG sites^[30]^. Notably, *Rv2966c* can also interact with histone proteins in the host cells^[22]^ and is a highly specific RNA MTase that methylates 16S rRNA of *M.tb *ribosome^[31]^. In addition to *Rv2966c*, other mycobacterial RNA MTases, such as *Rv3919c*,* Rv1694*, and* Rv1988*, are another group of mycobacterial MTases responsible for the methylation of certain nucleotides of rRNA. In turn, they affect various mechanisms in particular resistance to antibiotics^[32]^. Most antibiotics target the ribosomes of bacterial cells, and changes in these sites induce antibiotic resistance^[33]^. We reported the significant upregulation of* Rv3919c* in INH^R^, RIF^R^, MDR, and XDR strains, as well as a mutation (T to G) in codon 341 in INH^R^ strain. The streptomycin resistance in *M.tb *has been demonstrated to be associated with mutations reported in the *Rv3919c* (*gidB*) gene, a mycobacterial 16S rRNA MTase. Deletion of the *Rv3919c* gene in *M.tb *pathogens alters rRNA methylation and disrupts the binding of streptomycin to 16S rRNA, resulting in streptomycin resistance^[34]^. We confirmed resistance to streptomycin in RIF^R^, MDR, and XDR strains that indicated *Rv3919c* over-expression. Also, a possible reason for upregulation in INH^R^ strain may be its detected mutation in our study.

In this study, we detected no mutation in *Rv1694* and *Rv1988* genes. The upregulation of *Rv1694* and downregulation of *Rv1988 *was observed only in sensitive strains. *Rv1694* (*tlyA*) induces capreomycin sensitivity, and mutation in the *Rv1694* gene is associated with the induction of capreomycin resistance^[35]^. *Rv1988 *is a macrolide-lincosamide-streptomycin resistance gene belonging to the erythromycin resistance rRNA methylase family^[36]^. Similar to *Rv2966c*, *Rv1988 *is multifunctional that can epigenetically affect host genes and target immune genes involved in defense against *M.tb*^[37]^. Surprisingly, in this study, we presented the overexpression of *Rv1988* only in the drug resistant isolates. Deletion of this gene has been shown to be associated with reduced intracellular survival and is considered as an important virulence factor in *M.tb*^[19]^. This report confirmed that some MTases (such as *Rv2966c* and *Rv1988*) were certainly virulence factors that are involved in hijacking of the host transcriptional machinery and dampening its defense during initial of TB infection^[37]^. The variation of virulence may be along with the increasing antibiotic resistance. Thus, in controlling the spread of antibiotic resistance, controlling the spread of virulence is essential^[38]^.

The findings of the current study suggested the overexpression of *Rv1392* in sensitive, RIF^R^, MDR, and XDR strains, interestingly highlighting the role of MTases and methyl transfer in the resistance and pathogenesis of *M.tb*^[37]^. A significant upregulation of *Rv1392 *has also been reported in *M.tb* in comparison to mutant mycobacterial strain^[39,40]^. We detected a mutation (G to A) in INH^R^ strain that may be the cause of its downregulation.

In this study, we reported the difference of MTases expression between resistant and sensitive strains of *M.tb*. Overexpression of Rv0645c, Rv2756c, Rv3263, and Rv2966c was detected in all sensitive and resistant isolates. In the sensitive strain, Rv1988 and Rv3919c decreased but Rv1694 increased. Rv1392 expression level also decreased in INH isolate. Our results can be extended to this finding that mycobacterial MTases and SAM synthetase gene expression are more often reported in sensitive strains. A better understanding of the complete effects of mycobacterial MTase within a particular *M.tb* strain requires more detailed subsequent studies to integrate the data and develop new diagnostic and therapeutic strategies.

## DECLARATIONS

### Acknowledgments

The authors are grateful to the personnel of Mycobacteriology and Pulmonary Research Department, Pasteur Institute of Iran (Tehran) for their assistance in this project. We tender our apologies to those authors whose deserving research was not cited in this manuscript.

### Ethical statement

Not applicable.

### Data availability

Data supporting this article are included within the article and supplementary file. 

### Author contributions

ST and MSZ: performed all the experiments; ST, AF, and GB: analyzed and interpreted data; AF, FV, and MK: read and approved the final manuscript; SDS and AF: designed and supervised clinical study, interpreted data, read and approved the manuscript.

### Conflict of interest

None declared.

### Funding/support

The current project was supported by a grant (no. B-9426) from the Pasteur Institute of Iran, Tehran, Iran. The authors have no other relevant financial involvement with any organization or financial conflict with the subject matter or materials discussed in the manuscript apart from those disclosed. 

## Supplementary materials

**Supplementary Table 1:** Amplification parameters of PCR reaction

**Cycle**	**Temperature °C**	**Time**	**Step**
1	94	3 min	Initial Denaturing
35	94	45 s	Denaturing
	54	45 s	Annealing
	72	45 s	Extension
1	72	10 min	Melting

**Supplementary Table 2:** Amplification parameters of real-time PCR reaction

**Melting**	**Cycling (40 cycle)**	**Holding**
95 °C/5 s 60 °C/60 s 95°C/1 s	95 °C/5 min 50 °C/30 s 72 °C/30 sec	95 °C/60 s
